# Cardiopulmonary Arrest After COVID-19 Vaccination: A Case Report

**DOI:** 10.7759/cureus.21141

**Published:** 2022-01-12

**Authors:** Waleed Sadiq, Madeeha Subhan Waleed, Phyllis Suen, Michel N Chalhoub

**Affiliations:** 1 Medicine, Staten Island University Hospital, New York, USA; 2 Internal Medicine, Ayub Medical College, Abbottabad, PAK; 3 Internal Medicine, Northwell Health, Staten Island, USA; 4 Pulmonology and Critical Care Medicine, Staten Island University Hospital, New York, USA

**Keywords:** coronavirus, covid-19, sars-cov-2 and covid-19, covid-19 outbreak, cardiopulmonary arrest, covid-19 vaccination

## Abstract

Coronavirus disease 2019 (COVID-19) emerged in Wuhan in 2019 and by far has affected the whole world, and many people have succumbed to the disease. Vaccination programs introduced around the globe are aiming to reduce morbidity, mortality, and disease spread. We report the case of a 59-year-old male who suffered from cardiopulmonary arrest post-COVID-19 mRNA booster vaccination with no history of any other cardiopulmonary disease. Association between myocarditis and mRNA COVID-19 vaccines have been previously reported. However, this is the first case of cardiopulmonary arrest post-COVID-19 booster vaccination. Further research and cases should be described to confirm if this relationship exists. We need further cases to find this temporal association as such cases can also increase vaccine hesitancy. However, vaccination-associated adverse events should be vigilantly monitored and evaluated from time to time as further reports emerge.

## Introduction

Coronavirus disease 2019 (COVID-19) emerged in Wuhan in 2019 and by far has affected the whole world, and many people have succumbed to the disease. Due to the massive impact of the disease, the need for treatment and prevention is of paramount importance. This led to the development of vaccines against SARS-CoV-2 to reduce the morbidity and mortality associated with the disease. Coronavirus can cause various cardiovascular manifestations in an affected individual [1,2]. However, various adverse effects after acquiring the COVID-19 vaccination have also been reported. Mass vaccination programs in the United States against the coronavirus started in mid-December 2020, and almost 508 million doses have been given so far. However, to date, no case of cardiopulmonary arrest post-COVID-19 mRNA vaccination has ever been reported. This condition has serious consequences and can lead to sudden death, and it should be addressed within time by cardiopulmonary resuscitation (CPR), cardioversion, or cardiac pacing [3]. We report the first case of cardiopulmonary arrest post-COVID-19 booster vaccination in a patient with no history of any cardiopulmonary disease.

## Case presentation

We report the case of a 59-year-old male with no significant past medical history. He received his third dose of COVID-19 mRNA booster dose at 11:00 am, performed his errands, and went home. At 6:00 pm, he was found unresponsive in the house by his neighbors who performed chest compressions, and EMT services were called. The ACLS protocol was followed, and return of spontaneous circulation was achieved after 15 minutes. He was then intubated and transported to the emergency department. The patient had no known cardiac issues previously and had no exercise limitation before this event. In the emergency department, his blood pressure was 100/60 mmHg, his pulse rate was 88 beats per minute, an endotracheal tube was in place, and his oxygen saturation was 80% on 100% oxygen with positive end-expiratory pressure (PEEP) of 5 cm water. His Glasgow Coma Scale score was 5. Chest examination showed bilateral crackles. The patient was sedated, and an arterial line was placed. His severe acute respiratory syndrome coronavirus 2 RNA test with polymerase chain reaction was negative, and his other viral infection panel was nonsignificant.

**Figure 1:**
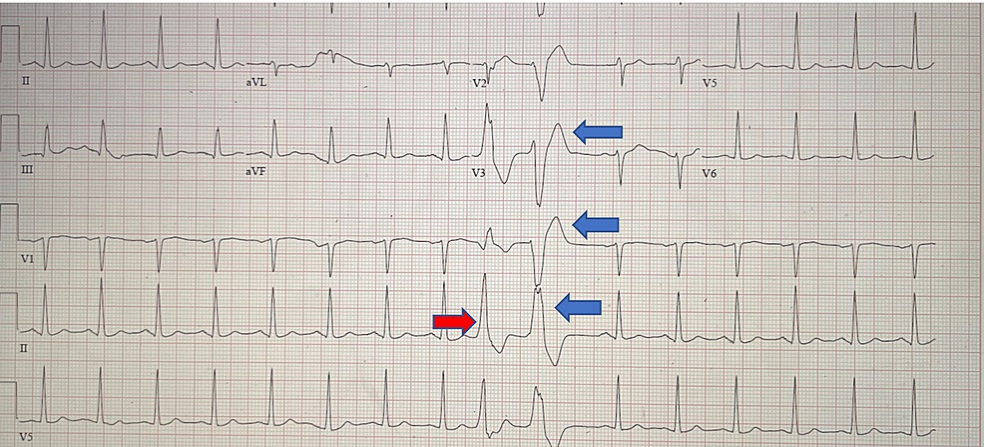
Electrocardiogram showing normal sinus rhythm with occasional premature ventricular complexes (blue arrows) and nonspecific ST and T wave abnormality (red arrow)

A 12‑lead electrocardiogram showed a normal sinus rhythm with occasional premature ventricular complexes and nonspecific ST and T wave abnormality, as shown in Figure 1.

There were no signs of anaphylaxis. Neuron-specific enolase was within range, and the Fungitell test along with other cultures was negative. Chest radiograph showed bilateral opacities. Arterial blood gas showed a profound metabolic acidosis along with lactic acidosis. The patient’s arterial blood gas is shown in Table 1.

**Table 1:** Arterial blood gases

Tests	Current Value	Reference Range
pH	7.15	7.35–7.45
Partial pressure of oxygen (PaO_2_)	80 mmHg	75–100 mmHg
Partial pressure of carbon dioxide (PaCO_2_)	56 mmHg	35–45 mmHg
Bicarbonate (HCO_3_)	20 mEq/L	22–26 mEq/L
Oxygen saturation (O_2_ sat)	80%	94%–100%

The patient was found to be profoundly hypoxic with an arterial partial pressure of oxygen of 80 mmHg on 100% oxygen, tidal volume of 420 mL, respiratory rate of 30 breaths per minute, and positive end-expiratory pressure (PEEP) of 10 cm water. His cardiac troponin levels were within the normal range. A transthoracic echocardiogram was performed and showed a normal ejection fraction (EF) with no regional wall abnormality. His complete metabolic panel is shown in Table 2.

**Table 2:** Complete metabolic profile

Tests	Current Value	Reference Range
Creatinine	0.8 mg/dL	0.76–1.27 mg/dL
Blood urea nitrogen (BUN)	13 mg/dL	6–20 mg/dL
Glomerular filtration rate	96 mL/minute/1.73	>59 mL/minute/1.73
Sodium	139 mmol/L	134–144 mmol/L
Potassium	4.1 mmol/L	3.5–5.2 mmol/L
Chloride	103 mmol/L	96–106 mmol/L
Carbon dioxide	23 mmol/L	20–29 mmol/L
Calcium	8.3 mg/dL	8.7–10.2 mg/dL
Glucose	172 mg/dL	65–99 mg/dL

Subsequently, the patient was kept in the ICU, and he remained hemodynamically stable. Over the course of days, the patient’s metabolic status and neurological function improved, but he could not be weaned off the ventilator, leading to tracheostomy and percutaneous endoscopic gastrostomy placement. The patient is currently undergoing rehabilitation and physical therapy in the hospital.

## Discussion

Our patient represents a case of favorable recovery post-cardiopulmonary arrest post-COVID-19 vaccination. This adverse effect has not been reported. We believe the cardiac arrest was most likely secondary to the COVID-19 booster vaccination. This is a diagnosis of exclusion, and after excluding all the potential causes of the cardiopulmonary arrest, the COVID-19 mRNA booster dose was the reasonable cause for the patient’s condition. The patient had no history of any cardiovascular conditions, and his COVID-19 PCR was negative as well. We are unaware of any other case of probable COVID-19-related cardiopulmonary arrest secondary to a booster dose of the COVID-19 vaccine. We report this case to increase awareness among healthcare providers and associated authorities to take this into account and continue to vigilantly monitor any side effects associated with the vaccination. However, vaccination is the ideal protection against the dreadful virus itself. Personal protective equipment (PPE) should always be worn by healthcare workers while assessing all patients in these times of uncertainty when the pandemic is rampant. Recently, the Centers for Disease Control and Prevention (CDC) Advisory Committee on Immunization Practices found an association between myocarditis and mRNA COVID-19 vaccines (Pfizer-BioNTech and Moderna) [4]. There is also the Vaccine Adverse Event Reporting System (VAERS), and a lot of cases of myocarditis and pericarditis have been reported after acquiring the mRNA COVID-19 vaccination [5]. Our patient also developed cardiopulmonary arrest after receiving the mRNA COVID-19 vaccine, and all the other possible causes of the cardiovascular event were excluded. Despite the various adverse events being reported, the COVID-19 vaccine remains the mainstay of disease control for coronavirus disease.

## Conclusions

Cardiopulmonary arrest with COVID-19 vaccination would be dependent on the manner of exclusion. Further research and cases should be described to confirm if this relationship exists. The mechanism is uncertain, and there is no specific test to determine the etiology as well. Hence, COVID-19 vaccine booster-related cardiopulmonary arrest is a diagnosis of exclusion. Early initiation of CPR in such patients can improve survival outcomes. COVID-19 vaccination is currently the only way available to control the ongoing pandemic and is vital to the prevention of disease transmission. The CDC and other appropriate and regulatory authorities should continue to monitor this and evaluate its risk as more reports emerge. Currently, vaccination is the only effective tool against the rampant spread of the virus. We need further cases to find this temporal association as such cases can also increase vaccine hesitancy. However, vaccination-associated authorities should be vigilantly monitoring the side effect profile, and vaccine risks and benefits should be reevaluated from time to time as further reports emerge.
